# Prevalence of undernutrition at admission and associated factors among children with cancer admitted to the University Hospital of Tangier: a cross-sectional study

**DOI:** 10.3389/fped.2026.1856098

**Published:** 2026-06-05

**Authors:** Amal Boumlik, Abdallah Oulmaati, Adil Najdi, Karima Sammoud, Yousra El Boussaadni

**Affiliations:** 1 Cancer and Chronic Diseases Laboratory, Faculty of Medicine and Pharmacy, University of Abdelmalek Essaâdi, Tangier, Morocco; 2 Mother and Child Department, University Hospital Mohamed VI, Tangier, Morocco; 3 Laboratory of Epidemiology and Public Health, Faculty of Medicine and Pharmacy, University of Abdelmalek Essaâdi, Tangier, Morocco

**Keywords:** associated factors, nutritional status, oncology, pediatrics, socioeconomic status

## Abstract

**Background/objectives:**

In pediatric oncology, undernutrition is a silent emergency. In developing countries, the situation is exacerbated by socio-economic insecurity and limited availability of specialized health services. The purpose of this research is to assess the prevalence of undernutrition and to analyze factors associated with undernutrition in children newly diagnosed with cancer.

**Methods:**

A total of 97 children, ranging in age from 6 months to 16 years, admitted for cancer to the hematology-oncology unit at the University Hospital of Tangier, were included in a cross-sectional study. Nutritional status was assessed using anthropometric indicators (BMI/A, MUAC/A) according to WHO standards. The analysis was based on a logistic regression model in order to identify factors associated with undernutrition.

**Results:**

The prevalence of undernutrition according to BMI/A was 35.1% [95% CI (25%; 45%)], and the factors associated with undernutrition were rural origin [OR=3.21; 95% CI: (1.15; 8.94); *p* = 0.02], and solid tumors [OR=2.31; 95% CI: (1.40; 3.80); *p* = <0.001]. In contrast, the prevalence of undernutrition was higher according to MUAC/A 44.3% [95% CI (34%; 54%)], the factors associated with undernutrition were rural origin [OR=3.40; 95% CI: (1.21; 9.94); *p* = 0.02], solid tumors [OR=2.02; 95% CI: (1.27; 3.20); *p* = 0.003], and low socioeconomic status [OR=6.10; 95% CI: (1.16; 31.82); *p* = 0.03]. No significant association was found between undernutrition and variables such as age or gender.

**Conclusion:**

Undernutrition is common in pediatric oncology, requiring the assessment of potential associated factors and the implementation of appropriate nutritional and educational protocols.

## 1 Introduction

Undernutrition is a major public health challenge, especially in regions where economic resources are limited and access to supportive care remains inadequate, further exacerbating its prevalence (1). In children with cancer, this nutritional disorder is a cause for concern, as it is not only a sign of the severity of the disease but also a factor influencing treatment tolerance, therapeutic response, and prognosis (2).

Despite advances in pediatric oncology, undernutrition remains one of the most common and underestimated complications (3). Its prevalence varies considerably depending on geographical context (4). According to a recent systematic review, the prevalence of undernutrition among children with cancer in 23 Low- and Middle-Income Countries (LMICs) ranges from 6.1% in China to 88.4% in South Africa, illustrating a marked disparity between socioeconomic contexts (5). Similarly, another study found a prevalence of undernutrition ranging from 0% to 65%, according to the nature of the tumor and the techniques employed to assess undernutrition (6).

Several studies have also shown that undernutrition at the diagnostic stage is accompanied by unfavorable complications, leading to an increase in the frequency of febrile neutropenia, infections, mucositis, increased intolerance to chemotherapy, and higher mortality rates (7–9). Poor nutritional status can also lead to alterations in the pharmacokinetics of anticancer drugs, modifying their metabolism, distribution, and elimination, and may also increase toxicity and compromise treatment efficacy (10).

Undernutrition in children with cancer results from several factors. It can be caused by the metabolic effects of the tumor or by cancer treatments, which frequently causes nausea, vomiting, and anorexia. The type and stage of the disease also influence nutritional status, as do age and difficult socioeconomic conditions, particularly in countries with limited resources (5, 11). All these factors combined make nutritional care essential for promoting better clinical outcomes and supporting the quality of life of these young patients (12). However, in Morocco, data on undernutrition among children with cancer remains limited. The few studies that have been conducted have focused mainly on assessing its prevalence and have not analyzed the factors associated with it. In the northern region, no study has yet addressed this issue. The Mohamed VI University Hospital in Tangier serves as the referral center for this region and treats almost all cases of pediatric cancer, providing a unique opportunity to assess the local situation.

In this context, the main objective of this study is to assess the prevalence of undernutrition at hospital admission and to analyze the factors associated with undernutrition in children with cancer treated at the hematology-oncology unit of the Tangier University Hospital. Based on existing scientific research, we hypothesize that undernutrition at admission may be associated with child's age, the type of cancer (solid vs. hematologic), and socioeconomic conditions. Indeed, the results of this study may guide decision-makers in the development of appropriate strategies and protocols to improve nutritional care for children treated for cancer.

## 2 Materials and methods

### 2.1 Type and setting of the study

This is a cross-sectional study that was performed in the pediatric hematology-oncology unit of the Mohamed VI University Hospital in Tangier, during the period spanning January 2025 to February 2026. This design was chosen to assess the prevalence of undernutrition at the time of diagnosis in children with cancer and to identify the associated factors.

### 2.2 Study population

All children aged 6 months to 16 years, residing in northern Morocco, admitted to the pediatric hematology-oncology unit of the Tangier University Hospital and with a confirmed diagnosis of cancer were included in the study (*n* = 97). The university hospital serves as the regional referral center for the entire northern region and treats all cancer cases, including transfers from other healthcare facilities in the region.

Patients were enrolled upon confirmation of diagnosis. Children with an unconfirmed diagnosis were excluded from the study. Sampling was exhaustive and consecutive.

### 2.3 Data collection methods

Information about participants was taken from patient records available on the hospital's electronic platform. The collection was based on a questionnaire for parents of children with cancer to gather sociodemographic information (child's age, gender, place of residence, parents’ education level, and occupation).

Anthropometric measurements were taken by healthcare staff in accordance with WHO standards. These measurements were then used to calculate anthropometric indicators (BMI/A and MUAC/A scores).

### 2.4 Variables studied

#### 2.4.1 Sociodemographic variables

Socioeconomic status (SES): assessed using Hollingshead's two-factor social status index, measured on the basis of parents’ occupation and educational attainment, and classified into three categories: low, medium, and high. This approach is widely used in scientific literature to measure socioeconomic status in public health (13, 14).

Geographic origin: noted from information collected through the questionnaire, coded based on the patient’s type of residence, urban or rural.

Gender and age group: collected from the questionnaire, gender was categorized as female/male and age into two groups: < 5 years and > 5 years.

#### 2.4.2 Clinical variables

Cancer type: collected from patients’ medical records, pediatric cancers in this study were subdivided into two main groups, hematologic malignancies and solid tumors.

#### 2.4.3 Anthropometric variables

BMI/A and MUAC/A: calculated to estimate the prevalence of undernutrition.

The selection of these variables is based on solid scientific evidence in the literature (3–5, 11).

##### 2.4.3.1 Primary outcome definition

The primary outcome was undernutrition, defined according to WHO anthropometric standards using BMI-for-age (BMI/A) and mid-upper arm circumference-for-age (MUAC/A) z-scores. A z-score < −2 standard deviations was considered indicative of undernutrition. These measures were used to estimate its prevalence and assess associated factors.

### 2.5 Assessment of nutritional and socioeconomic status

Nutritional status was assessed using anthropometric measurements, such as BMI and MUAC.

Weight of children over 2 years of age was measured using a standing scale (SECA model P786, accuracy ± 100 g). For children under 2 years of age, weight was obtained using a calibrated baby scale (SECA model P354-T417) (accuracy ±100 g), with the child lying down, without clothes or lightly clothed. Children who were able to stand were weighed directly on the scale, barefoot and lightly clothed. Height of children over two years was measured using a fixed vertical stadiometer, in a standing position with their head straight. Length of children under two years of age was measured in the supine position using a horizontal infantometer. Each measurement (weight and height) was taken twice consecutively, and the calculation was based on the average of the two values. The BMI/A z-score was determined using the WHO growth references. A BMI/A z-score < −2 standard deviations was considered indicative of undernutrition, according to WHO recommendations (15, 16). To calculate BMI/A z-scores, we used WHO calculation soft-ware: WHO Anthro was used for children under 5 years of age, while WHO Anthro Plus was used for children and adolescents aged 5–19 years, in accordance with the 2006 and 2007 WHO growth standards (15, 16).

MUAC was measured at mid-left arm, between the acromion and olecranon, with the child sitting or lying down and the arm relaxed alongside the body. A non-stretchable MUAC tape was used for all measurements. Each measurement was taken twice and the average value was recorded. For children aged 6–59 months, classification thresholds followed WHO reference standards (15, 17). A MUAC/A z-score < −2 standard deviations were considered indicative of undernutrition, while a MUAC/A z-score ≥ −2 standard deviations indicated normal nutritional status (no undernutrition). The MUAC/A z-score was calculated using WHO Antro software (15).

For children aged 5–16 years, classification thresholds were established based on growth curves for the MUAC/A z-score that comply with WHO growth standards, with undernutrition defined as a MUAC/A z-score < −2 standard deviations, However, a MUAC/A z-score ≥ −2 standard deviations indicated normal nutritional status (no under-nutrition) (18). The MUAC/A z-score was calculated using the references established by Mramba et al. (18), using the validated online tool PediTools MUAC-for-age Calculator (19).

Socioeconomic status was calculated based on the parents’ occupation and level of education, as determined by a questionnaire based on Hollingshead's two-factor social status index. To obtain an SES score for each child, we combined the parents’ level of education and occupation. Each level of education was rated from 1 to 7 (from 1 = illiterate to 7 = doctorate/higher professional training), and from 1 to 9 for occupation (from 1 = no occupation to 9 = senior executives and liberal professions). Each parent's SES score was then calculated by multiplying educational attainment by 3 and occupation by 5. The child's final SES score was obtained by calculating the average of the two parents’ scores. For children with only one parent, the score of the parent present was used as the child's score. SSI scores ranged from 8 to 66. Scores between 8 and 29 correspond to a low SES, scores between 30 and 40 reflect an average SES, while scores above 40 indicate a high SES (13, 14).

### 2.6 Statistical analysis

Data processing, organization, and presentation of results were performed using SPSS statistical software version 27 and Excel version 2016. Qualitative variables of the participants were reported as percentages, and quantitative variables were presented as mean, standard deviation, median and IQR. Bivariate analysis of associations between nutritional status (according to MUAC/A or BMI/A) was performed using the chi-square test, applied for the analysis of independent variables.

For the multivariable logistic regression analyses, results were reported as odds ratios (ORs) with 95% confidence intervals (CIs), and statistical significance was set at *p* < 0.05. Variable selection was primarily based on prior literature and a predefined conceptual framework, complemented by statistical considerations when appropriate. To limit overfitting, the number of predictors included in the models was determined according to the events-per-variable (EPV) rule, with a minimum of 10 events per variable. Multicollinearity was assessed using variance inflation factors (VIF). Model fit was evaluated using the Hosmer–Lemeshow goodness-of-fit test. Influential observations were examined using Cook's distance. Internal validation was performed using bootstrap resampling with 1,000 iterations to obtain bias-corrected and accelerated (BCa) confidence intervals.

### 2.7 Ethical considerations

The study received ethical approval from the committee of [the Faculty of Medicine and Pharmacy of Tangier] under number [AC148MA/2025], and approval. Confidentiality and informed consent from parents were guaranteed in this study.

## 3 Results

### 3.1 Sociodemographic and clinical characteristics and nutritional status of children with cancer

This study included 97 children with cancer. The children's ages ranged from 6 to 187 months, or [0.5 years; 15.6 years]. The mean age was 93.8 months (7.8 years), with a standard deviation of 52.2 months (4.3 years). The median age was 97 months (8.1 years). The population consisted of *n* = 53 boys (54.6%) and *n* = 44 girls (45.4%), corresponding to a boy/girl ratio of 1.2. The mean socioeconomic status score of the participants, calculated from the mean scores of both parents, was 20.3 (standard deviatio*n* = 8.3). The range of scores varied from 8 to 37.5. Among the children included, 28.9% (*n* = 28) were under 5 years of age, while 71.1% (*n* = 69) were 5 years of age or older. The BMI/A z-scores had a mean of −1.2 ± 1.4, with a median of −1.1 and an interquartile range (IQR) of 2.5, while the MUAC/A z-scores had a mean of −1.6 ± 1.5, a median of −1.6, and an IQR of 2.5 (Table 1).

**Table 1:** Sociodemographic and anthropometric characteristics of children with cancer.

Variables	Indicators	Values
Age (Years)	Mean ± Standard deviation	7.8 ± 4.3
	Range (min–max)	0.5–15.6
Sex	Male *n* (%)	53 (54.6%)
	Female *n* (%)	44 (45.4%)
	M/F ratio	1.2
Mean score of SES	Mean ± Standard deviation	20.3 ± 8.3
	Range (min–max)	8–37.5
Weight (kg)	Range (min–max)	6.3– 67
Height (cm)	Range (min–max)	69–174
MUAC (cm)	Range (min–max)	10–27
BMI/A z-scores	Median	−1.1
	IQR	2.5
MUAC/A z-scores	Median	−1.6
	IQR	2.5

SES, socioeconomic status; MUAC, mid-upper arm circumference; BMI/A, body mass index for age; MUAC/A, mid-upper arm circumference for age; IQR, interquartile range.

BMI/A or MUAC/A z-score < −2: undernourished (yes). Z-score ≥ −2: undernourished (no); SES scores: 8–29 = low, 30–40 = average, >40 = high.

BMI/A, body mass index for age; MUAC/A, mid-upper arm circumference for age.

Solid tumors accounted for 52.6% (*n* = 51) of cases, while hematologic malignancies represented 47.4% (*n* = 46). Among solid tumors, neuroblastoma was the most frequent (*n* = 14, 27.5%), followed by bone tumors (*n* = 11, 21.6%) and renal tumors (*n* = 10, 19.6%). Other tumor types included soft tissue tumors (*n* = 6, 11.8%), germ cell tumors (*n* = 3, 5.9%), and hepatic tumors, cerebral tumors, and carcinomas (each *n* = 2, 3.9%), while retinoblastoma was less frequent (*n* = 1, 2%). Among hematologic malignancies, leukemia predominated (*n* = 33, 71.7%), followed by lymphoma (*n* = 13, 28.3%).

**Figure 1:**
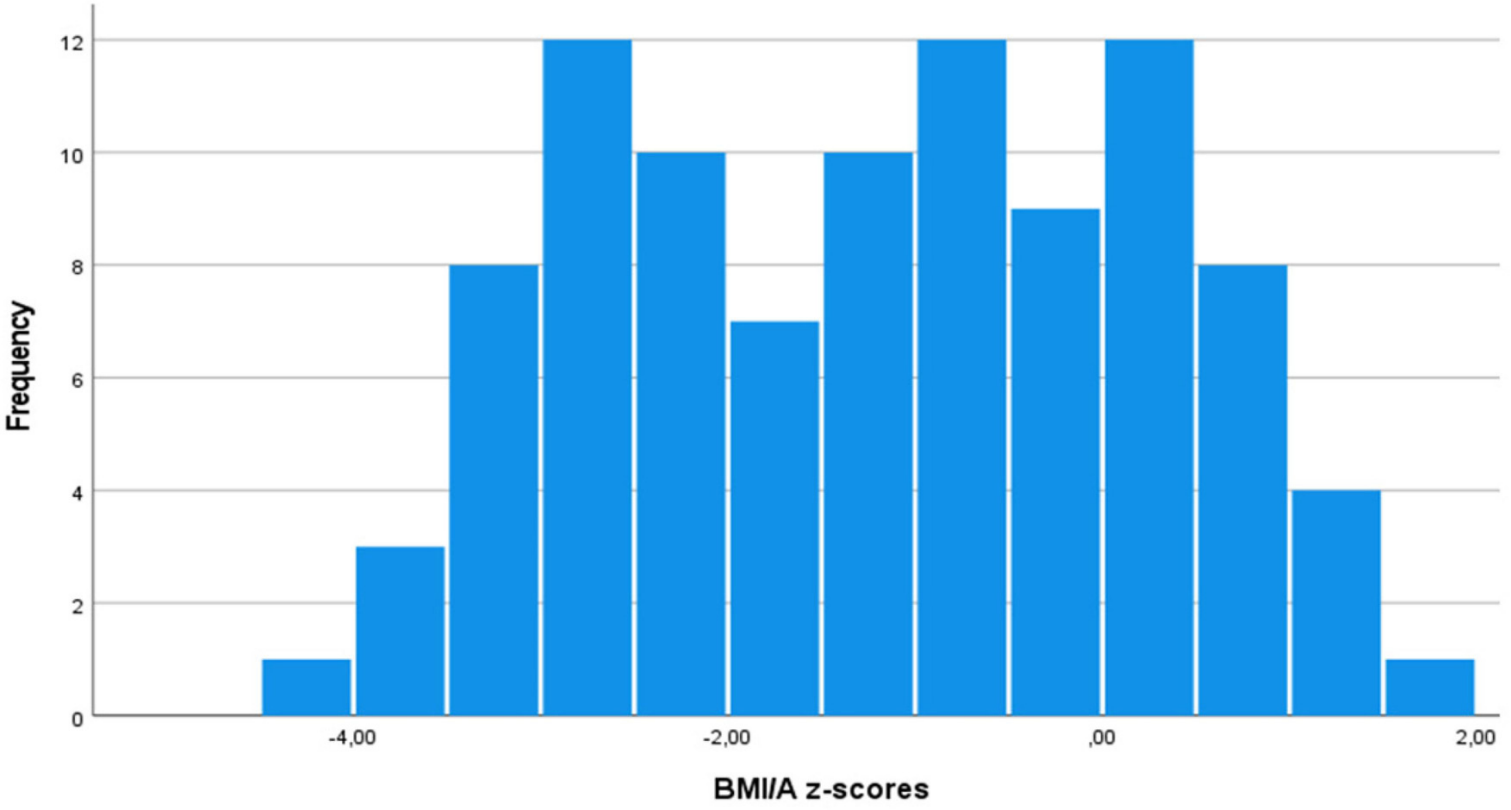
Distribution of BMI/A Z-scores in children with cancer.

**Figure 2:**
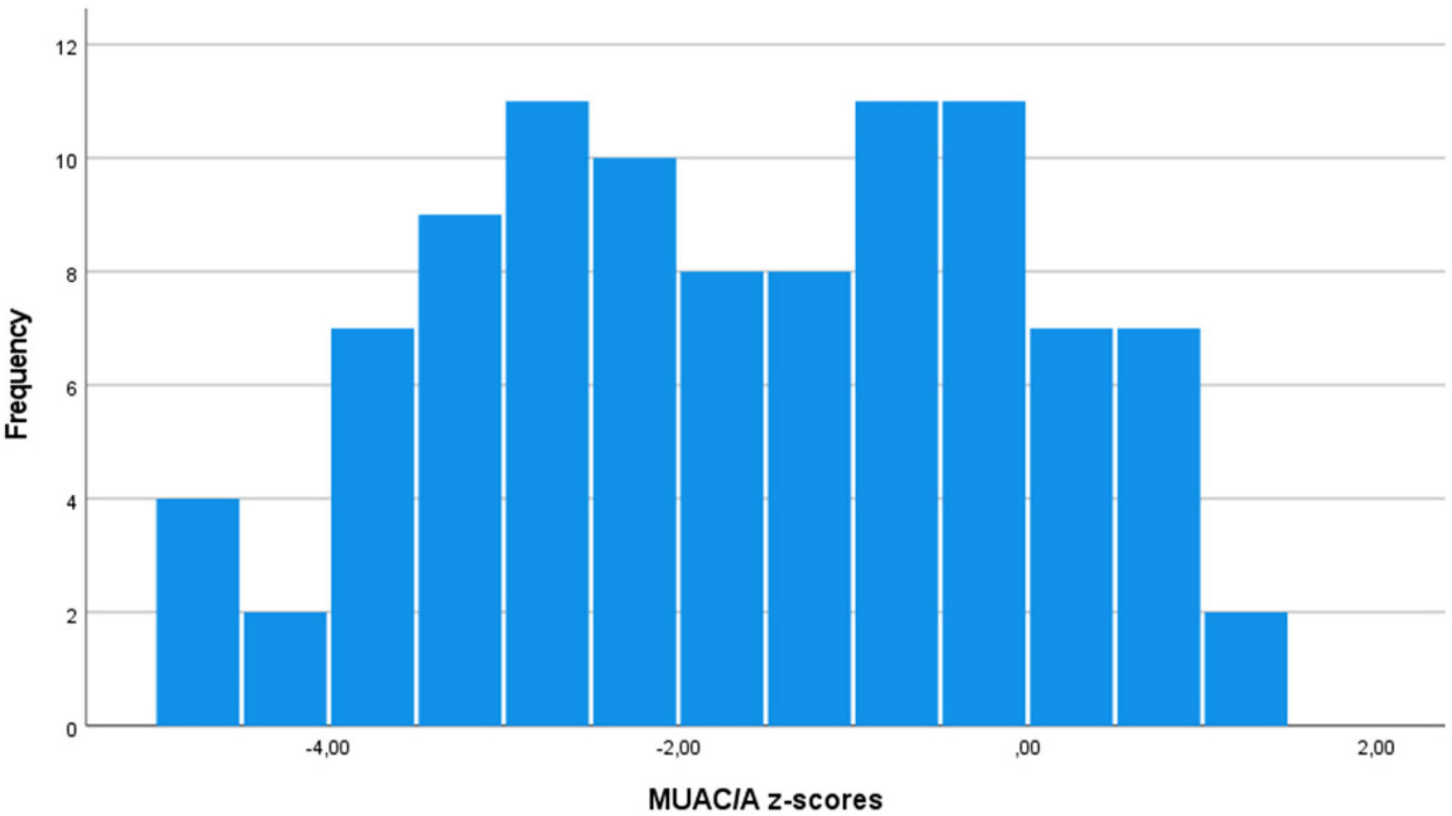
Distribution of MUAC/A Z-scores in children with cancer.

The distribution of BMI/A z-scores was fairly symmetrical around zero, with frequencies ranging from 1 to 12 over intervals between approximately −4.0 and −2.0. Most children had z-scores between −3.0 and 1.0, indicating a wide variation in nutritional status based on BMI. Similarly, the distribution of MUAC/A z-scores was roughly symmetrical, but with a slightly higher frequency at lower z-score intervals, ranging from approximately −4.0–2.0. The majority of children had z-scores between −3.5 and −0.5, reflecting the variability in nutritional status based on upper arm circumference (Figures 1, 2).

The BMI/A assessment identified 34 children [35.1%, 95% CI(25%; 45%)] undernourished. According to the MUAC/A, 43 children [44.3%, 95% CI (34%; 54%)] were undernourished (Table 2).

**Table 2:** Prevalence of undernutrition according to BMI/A and MUAC/A.

Indicators	Categories	n	%
BMI/A	Undernourished/Yes Undernourished/No	34 63	35.1 64.9
MUAC/A	Undernourished/Yes Undernourished/No	43 54	44.3 55.7

### 3.2 Bivariate analysis of factors associated with undernutrition in children with cancer using BMI/A and MUAC/A

According to MUAC/A, undernutrition was significantly associated with rural residence *n* = 18 (69.2%) compared to those from urban areas *n* = 25 (35.2%) (*p* = 0.003). A significant association was also observed between undernutrition and cancer type, with children with solid tumors having a prevalence of 58.8%, compared to 28.3% in those with malignant blood disorders (*p* = 0.002). Similarly, low socioeconomic status was significantly associated with undernutrition, with a *p*-value of 0.01. However, no association was found with gender (*p* = 0.84) or age group (*p* = 0.28) (Table 3).

**Table 3:** Bivariate analysis of factors associated with undernutrition in children with cancer according to MUAC/A.

Factors	Category	n (%)	Undernourished	Undernourished	*p*-value
			(No) *n* (%)	(Yes) *n* (%)	
Sex	Male	53 (54.6)	29 (54.7)	24 (45.3)	0.84
	Female	44 (45.4)	25 (56.8)	19 (43.2)	
Age group	Over 5 years	69 (71.1)	36 (52.2)	33 (47.8)	0.28
	under 5 years	28 (28.9)	18 (64.3)	10 (35.7)	
Residence	Urban	71 (73.2)	46 (64.8)	25 (35.2)	0.003
	Rural	26 (26.8)	8 (30.8)	18 (69.2)	
Socio-economic status	Middle	14 (14.4)	12 (85.7)	2 (14.3)	0.01
	Low	83 (85.6)	42 (50.6)	41 (49.4)	
Type of cancer	Hematologic malignancies	46 (47.4)	33 (71.7)	13 (28.3)	0.002
	Solid tumors	51 (52.6)	21 (41.2)	30 (58.8)	

According to BMI/A, undernutrition was also significantly associated with rural residence *n* = 15 (57.7%) than among children from urban areas 19 (26.8%) (*p* = 0.005). A significant difference was also observed according to cancer type (*p* =< 0.001), with a higher prevalence among children with solid tumors (51%). However, no statistically significant relationship was observed with gender, age group, or socioeconomic status (*p* > 0.05) (Table 4).

**Table 4:** Bivariate analysis of factors associated with undernutrition in children with cancer according to BMI/A.

Factors	Category	n (%)	Undernourished (No) *n* (%)	Undernourished (Yes) *n* (%)	*p*-value
Sex	Male	53 (54.6)	33 (62.3)	20 (37.7)	0.54
	Female	44 (45.5)	30 (68.2)	14 (31.8)	
Age group	Over 5 years	69 (71.1)	44 (63.8)	25 (36.2)	0.70
	Under 5 years	28 (28.9)	19 (67.9)	9 (32.1)	
Residence	Urban	71 (73.2)	52 (73.2)	19 (26.8)	0.005
	Rural	26 (26.8)	11 (42.3)	15 (57.7)	
Socio-economic status	Middle	14 (14.4)	12 (85.7)	2 (14.3)	0.08
	Low	83 (85.6)	51 (61.4)	32 (38.6)	
Type of cancer	Hematologic malignancies	46 (47.4)	38 (82.6)	8 (17.4)	< 0.001
	Solid tumors	51 (52.6)	25 (49)	26 (51)	

### 3.3 Multivariate analysis of factors associated with undernutrition in children with cancer according to BMI/A and MUAC/A

Factors independently associated with undernutrition according to MUAC/A were identified using multivariate logistic regression analysis. After adjusting for significant variables in bivariate analysis, three remained significantly associated with undernutrition: low socioeconomic status [OR=6.10; 95% CI: (1.16; 31.82); *p* = 0.03], solid tumors [OR=2.02; 95% CI: (1.27; 3.20); *p* = 0.003], and rural origin [OR=3.40; 95% CI: (1.21; 9.94); *p* = 0.02]. However, multivariate analysis by logistic regression according to BMI/A. showed that only two factors were significantly associated with undernutrition: rural origin [OR=3.21; 95% CI: (1.15; 8.94); *p* = 0.02] and solid tumors [OR=2.31; 95% CI: (1.40; 3.80); *p* = < 0.001] (Table 5).

**Table 5:** Multivariate analysis of factors associated with undernutrition in children with cancer according to BMI/A and MUAC.

Factor	BMI/A OR (95% CI)	*p*-value	MUAC/A OR (95% CI)	*p*-value
Solid tumor types	2.31 (1.40–3.80)	<0.001	2.02 (1.27–3.20)	0.003
Rural residence	3.21 (1.15–8.94)	0.02	3.40 (1.21–9.94)	0.02
Low socioeconomic status	3.81 (0.71–20.18)	0.11	6.10 (1.16–31.82)	0.03

BMI/A, body mass index for age; MUAC/A, mid-upper arm circumference for age; CI, confidence interval.

No evidence of severe multicollinearity was observed (maximum condition index = 12.5). A few observations showed moderate influence according to Cook's distance (> 0.5), but sensitivity analyses excluding these observations yielded similar estimates. Bootstrap resampling (1,000 iterations) confirmed the stability of the regression coefficients, although socioeconomic status exhibited wider confidence intervals, indicating greater uncertainty for this variable. Finally, the Hosmer–Lemeshow test indicated an adequate model fit (*p* > 0.05).

## 4 Discussion

### 4.1 Prevalence of undernutrition in pediatric cancer patients and comparison of anthropometric indicators

In our study, the prevalence of undernutrition among children with cancer was 44.3% according to MUAC/A and 35.1% according to BMI/A. These rates are within the range of undernutrition prevalence observed in LMICs, as reported in a systematic review based on the results of eight studies conducted in Africa involving 742 children, which revealed a prevalence range of 9.0% to 78.9% (5). Similarly, a meta-analysis conducted in sub-Saharan Africa estimated an overall prevalence of undernutrition of 41.34% in children diagnosed with cancer (4).

The prevalence of undernutrition observed in our study is comparable to that reported in other LMICs. The study conducted in India, involving 1,042 children with cancer hospitalized in Delhi, revealed a prevalence of undernutrition of 39.7% at the time of diagnosis, which is very similar to the findings of our study (20).

Furthermore, this study observed that assessing undernutrition using MUAC/A detected a higher prevalence of undernutrition than BMI/A based on weight and height. This difference can be explained by their methodological properties and the pathophysiological conditions they reflect. MUAC is a sensitive anthropometric indicator that reflects acute undernutrition resulting from the loss of muscle mass and peripheral lean tissue, which occurs early in children undergoing treatment, regardless of total weight or height (21, 22). BMI/A, on the other hand, reflects overall undernutrition, which can be influenced by edema and large tumors, particularly intra-abdominal tumors (23–25). Consequently, the prevalence of undernutrition could be underestimated when using BMI/A alone. These findings corroborate the results of a study conducted in Casablanca on the nutritional status of children with malignant tumors, which revealed a prevalence of undernutrition ranging from 20% to 50% depending on the anthropometric indicator used (26). Shah et al. (27) reported in their study a prevalence of undernutrition ranging from 38% to 76% in children with cancer, depending on the anthropometric indicator used (BMI/A, MUAC/A) (27).

### 4.2 Type of cancer associated with undernutrition

In the present study, solid tumors represented a slightly larger proportion of cases compared with hematologic malignancies, with neuroblastomas, bone tumors, and renal tumors being predominant.

In addition, a significant association was noted between undernutrition and solid tumors. This result suggests that children with solid tumors are at higher risk of undernutrition than those with hematologic malignancies. This result is consistent with the findings of several studies that have shown that tumor location directly influences the nutritional status of children with cancer. A cohort study evaluating the prevalence of undernutrition and its impact on outcomes in children with cancer noted that in South Africa, 21.2% of children with solid tumors were undernourished, compared with 7.7% of children with hematologic cancers (28). Furthermore, another study conducted in India found that the prevalence of malnutrition was significantly associated with the type of cancer (solid tumors) with a *p*-value of 0.018 (29).

Solid tumors are often associated with significant metabolic changes, chronic inflammation, and increased resting energy expenditure, which promotes muscle mass breakdown and lean tissue loss (23, 30). In addition, this can also be explained by the fact that most solid tumors develop chronically and that, in LMICs, patients tend to seek medical attention late. This negatively affects the child's nutritional status due to the metabolic demands of the tumor, to the detriment of the child's growth (31).

### 4.3 Sociodemographic factors associated with undernutrition

Socioeconomic status may indirectly influence undernutrition, particularly through access to healthcare, education, and psychological support. Several studies have shown that in LMICs, socioeconomic insecurity remains a factor in nutritional vulnerability, although its influence is partly linked to the action of other factors (20).

In fact, an association was observed between SES and undernutrition. The same conclusion was reached by a study conducted in Guatemala on the link between the nutritional status of children with cancer and socioeconomic status, which showed that SES was associated with the nutritional status of children with cancer, the lower the SES, the greater the nutritional vulnerability, with a highly significant association (*p* < 0.0001) (32).

In our study, rural origin emerged as a factor significantly associated with undernutrition according to BMI/A and MUAC/A indicators. Srivastava et al. reported that rural origin was significantly associated with undernutrition in children with cancer (*p* ≤ 0.001) (29). This observation is part of a broader context of nutritional vulnerability among children living in rural areas. Indeed, a study on the nutritional status of children according to origin (rural or urban) Based on data from demographic and health surveys conducted in 35 developing countries, the study noted that children from rural areas are more likely to have high rates of undernutrition. The study linked this finding to several factors, namely limited access to specialized healthcare facilities (33).

However, our study did not reveal any significant association between the sex and nutritional status of the children at the time of cancer diagnosis. These results suggest that demographic factors such as sex do not appear to directly influence the prevalence of undernutrition in this population. Several previous studies have also reported similar results. A male predominance in the male/female ratio is frequently observed, as is the case in our study. However, from a statistical point of view, the association between children's gender and their nutritional status remains inconclusive (29, 34, 35). Furthermore, in our study, age do not appear to be associated with nutritional status. This finding corroborates the results of Katabalo et al., which show no statistically significant association between the nutritional status of children with cancer and age (11). However, some studies have shown a relationship between undernutrition and children under 5 years of age (36, 37), while others have found an association mainly in children over 5 years of age (5, 34, 38). The divergence observed in the literature could be explained by different socioeconomic and demographic contexts, different methodologies, particularly the anthropometric indicators used or the age groups selected.

### 4.4 Recommendations and practical implications

Given these results, it is recommended that a systematic nutritional assessment be carried out at the time of cancer diagnosis in children, using complementary anthropometric indicators such as BMI for age and MUAC for age, to enable early detection of undernutrition (39). These two indicators should be applied in parallel, as they capture different and complementary dimensions of nutritional status. BMI-for-age is more appropriate for identifying chronic undernutrition, whereas MUAC-for-age is more sensitive to acute malnutrition and rapid changes in body composition, particularly in children with edema or rapid weight loss (23–25, 39). Specifically, a BMI-for-age z-score < −2 should prompt a thorough evaluation for chronic undernutrition, while a MUAC-for-age z-score < −2 should prompt immediate management in cases of acute undernutrition or in the presence of edema (15, 16, 18). MUAC is particularly useful for children experiencing rapid weight loss or edema, whereas BMI-for-age is more appropriate for detecting long-term nutritional deficits (21–25). Regular nutritional monitoring throughout treatment should also be implemented to help prevent deterioration and allow timely correction of deficiencies (12).

From a clinical perspective, nutritional screening and monitoring should be integrated into pediatric oncology care to support timely and appropriate interventions. Individualized nutritional support could be prioritized for children at higher potential risk, including those with solid tumors, those from low socioeconomic backgrounds, and those living in rural areas, as it may improve treatment tolerance and reduce complications and mortality (32, 40).

At the health system level, strengthening healthcare workers’ competencies in pediatric oncology nutrition remains essential. In LMICs, this could be achieved by integrating nutritional care into national cancer control programs through context-adapted guidelines and protocols, as well as family centered nutrition education programs aimed at supporting dietary management during treatment (3, 5, 40).

From a research perspective, prospective studies are needed in LMICs, particularly in North Africa, to better document the nutritional status of children with cancer. These studies should include biological and clinical indicators and also consider age related vulnerabilities, particularly in children under 2 years and adolescents, in order to identify populations at highest risk of undernutrition and to guide appropriate intervention strategies (12).

### 4.5 Strengths and limitations of the study

This study presents several strengths and limitations. Nutritional status was assessed at the time of diagnosis, providing a reliable baseline description of children with cancer (39, 41). The combined use of BMI-for-age (BMI/A) and MUAC-for-age (MUAC/A) is a methodological strength, as these indicators are complementary in capturing different dimensions of undernutrition (42). Statistical analyses, including bivariate and multivariate models, allowed the exploration of associations between nutritional status and selected sociodemographic and clinical variables, providing descriptive evidence useful for clinical interpretation. This study contributes data on the nutritional status of children with cancer in northern Morocco, a setting where evidence remains limited. These findings should be interpreted in the context of the study's limitations, taking into account the monocentric design and the relatively limited sample size. Several limitations should be acknowledged. The study was based on a relatively limited sample size from a single referral center, which may affect the representativeness of the findings. Key potential confounding factors, including disease stage, clinical severity, and inflammatory markers, were not available and could not be controlled for, which may have influenced the observed associations. Due to the cross-sectional design, no causal relationships can be inferred between nutritional status and the studied variables. Nutritional assessment was based exclusively on anthropometric indicators (BMI/A and MUAC/A z-scores), which do not capture the full complexity of nutritional alterations in pediatric oncology. However, these indicators are recommended by the WHO and widely used in similar resource-limited settings, supporting their appropriateness for descriptive epidemiological analysis.

## 5 Conclusions

Undernutrition appears to be common among children with cancer in northern Morocco. Children with solid tumors, as well as those from low socioeconomic backgrounds or rural areas, may be at increased risk of undernutrition. No clear association was observed with age or gender. These findings underscore the importance of early, context-specific nutritional assessment to support optimal management and follow-up in this vulnerable population.

## Data Availability

The datasets presented in this article are not readily available due to the inclusion of patient data and confidentiality considerations, but may be made available by the corresponding author upon reasonable request and subject to ethical approval.
